# Transcriptome analysis of porcine PBMCs reveals lipopolysaccharide-induced immunomodulatory responses and crosstalk of immune and glucocorticoid receptor signaling

**DOI:** 10.1080/21505594.2021.1948276

**Published:** 2021-07-21

**Authors:** Zhiwei Li, Nares Trakooljul, Frieder Hadlich, Siriluck Ponsuksili, Klaus Wimmers, Eduard Murani

**Affiliations:** Institute of Genome Biology, Leibniz Institute for Farm Animal Biology (FBN), Dummerstorf, Germany

**Keywords:** Transcriptome, porcine pbmcs, lipopolysaccharide, immune signaling, dexamethasone, glucocorticoid receptor signaling

## Abstract

The current level of knowledge on transcriptome responses triggered by endotoxins and glucocorticoids in immune cells in pigs is limited. Therefore, in the present study, we treated porcine peripheral blood mononuclear cells (PBMCs) with lipopolysaccharide (LPS) and dexamethasone (DEX) separately or combined for 2 hours. The resultant transcriptional responses were examined by mRNA sequencing. We found that the LPS treatment triggered pronounced inflammatory responses as evidenced by upregulation of pro-inflammatory cytokines, chemokines, and related signaling pathways like NF-κB. Concurrently, a series of downregulated pro-inflammatory and upregulated anti-inflammatory molecules were identified. These are involved in the inhibition of TLR, NF-κB, and MAPK cascades and activation of signaling mediated by Tregs and STAT3, respectively. These findings suggested that LPS initiated also an anti-inflammatory process to prevent an overwhelming inflammatory response. The transcriptome responses further revealed substantial crosstalk of immune responses and glucocorticoid receptor (GR) signaling. This was apparent in four aspects: constitutive inhibition of T cell signaling by DEX through a subset of genes showing no response to LPS; inhibition of LPS-induced inflammatory genes by DEX; attenuation of DEX action by LPS paralleled by the regulation of genes implicated in cytokine and calcium signaling; and DEX-induced changes in genes associated with the activation of pro-inflammatory TLR, NF-κB, iNOS, and IL-1 signaling. Consequently, our study provides novel insights into inflammatory and GR signaling in pigs, as well as an understanding of the application of glucocorticoid drugs for the treatment of inflammatory disorders.

## Introduction

Glucocorticoids (GCs) are considered to be the most potent and effective anti-inflammatory drugs in both human and veterinary medicine [1]. Immunomodulation by GCs is mediated primarily by the glucocorticoid receptor (GR), a ligand-inducible transcription factor (TF) of the nuclear receptor superfamily. GR signaling plays a vital role in many biological processes, such as cell proliferation and metabolic regulation [2]. It is generally believed that the anti-inflammatory action of GR is conferred by its monomeric form through transrepression of other, pro-inflammatory, TFs such as NF-κB, AP-1, IRF3, and T-bet. In contrast, GR dimer-dependent transactivation of genes involved in glucose and lipid metabolism is associated with undesirable side effects on metabolic homeostasis [2]. However, there is increasing evidence suggesting that the dimeric form is crucial in GR-mediated anti-inflammatory action, which is determined by a series of GC-inducible anti-inflammatory molecules, including TSC22D3, KLF2, and DUSP1 [3]. On the other hand, immune mediators like cytokines exert considerable influence on GR signaling [4,5]. These findings emphasize the complexity and diversity of GR signaling and its function in controlling inflammation. It is vital that comprehensive research is undertaken to further explore the regulation of GR signaling and its crosstalk with immune pathways.

The current use of GC-based drugs in pigs relies mainly on findings from human studies since knowledge of the effects of these drugs in farm animals is relatively lacking [6]. However, the distinct potency and pharmacokinetics of GC-based drugs, such as dexamethasone (DEX), in pigs, calls for deeper research in this field [7]. Moreover, pigs are comparatively more vulnerable to lipopolysaccharide (LPS) [8]. LPS administration induces a pronounced inflammatory response in pigs, alongside behavioral and physiological changes; most of these transformations are attenuated by co-administration of DEX [9,10]. We have previously shown that short-term treatment by DEX regulates a large number of genes involved in inflammatory responses in the porcine liver even in the absence of immune stimuli [11]. This finding accentuates the substantial role of GCs and GC-based drugs in the immunomodulation in pigs. However, how acute activation of GR signaling by short-term exposure to GCs orchestrates responses in the presence and absence of immune stimuli in porcine immune cells is still poorly explored.

This study aims to investigate GR signaling and inflammatory responses, and to establish their interplay in porcine immune cells. To this end, porcine peripheral blood mononuclear cells (PBMCs) were treated with either vehicle (CON), DEX, LPS, or LPS+DEX for 2 hours to mimic acute inflammation and activation of GR signaling. The corresponding transcriptome responses were explored using mRNA sequencing; in addition, a range of different bioinformatics tools were employed to obtain a holistic overview of the events. The findings of this study will facilitate improved and informed application and development of GC-based drugs, and will also offer an insight into how stress – via the induction of natural GCs – modulates the immune system and influences animal health. These are important, foundational steps leading toward the successful application of the One Health concept [12].

## Materials and methods

### Sample collection

German Landrace pigs used to collect samples were raised until slaughter age (mean = 170 days) under standardized conditions at the experimental pig farm of the Leibniz Institute for Farm Animal Biology (Dummerstorf, Germany) in accordance with the German Law of Animal Protection.

PBMCs were isolated from whole blood as previously described [13]. Briefly, trunk blood was collected into pre-chilled tubes containing EDTA during exsanguination in the context of regular slaughter procedures, taking place in the morning. The blood samples were then centrifuged on a Histopaque-1077 density gradient (Sigma–Aldrich, Taufkirchen, Germany) to attain a layer of PBMCs, according to the manufacturer’s instructions. The isolated PBMCs were stored in liquid nitrogen with 90% fetal bovine serum (FBS; PAN Biotech, Aidenbach, Germany) and 10% DMSO until required.

## *In vitro* LPS and DEX challenge

PBMCs taken from whole blood samples of 24 pigs (12 males, 12 females) were used for treatment assays as previously described with modifications [14]. First, cells were thawed and washed with RPMI 1640 medium (Biochrom, Berlin, Germany). Subsequently, cells were resuspended in cell culture medium (RPMI 1640 medium supplemented with 10% FBS, 2 mmol/l L-glutamine (Biochrom, Berlin, Germany), 100 U/ml penicillin, and 100 μg/ml streptomycin (Sigma–Aldrich, Taufkirchen, Germany)) and adjusted to 6 × 10^6^ cells/ml. PBMCs from each individual were divided into four treatment groups and seeded in 24-well plates at 3 × 10^6^ cells/well, followed by overnight incubation at 37°C with 5% CO_2_. DEX and LPS stock solutions were prepared in ethanol (25 mM) and PBS (1 mg/ml), respectively, and diluted in cell culture medium to the required concentration as needed. The four groups were treated with either vehicle (CON; cell culture medium + corresponding volume of ethanol + corresponding volume of PBS), DEX (Sigma–Aldrich; final concentration 5 nM (≈ 2 ng/ml) in cell culture medium + corresponding volume of PBS), LPS (*Escherichia coli* O111: B4; Sigma–Aldrich; final concentration 10 μg/ml in cell culture medium + corresponding volume of ethanol), or LPS (10 μg/ml) + DEX (5 nM), respectively. All the samples were treated for 2 hours at 37°C with 5% CO_2_. Afterward, cells were collected for RNA extraction.

## RNA extraction and mRNA sequencing

Total RNA was extracted using TRI reagent (Sigma–Aldrich, Taufkirchen, Germany) and purified with the RNA Clean & Concentrator-25 Kit (Zymo Research, Freiburg, Germany), in accordance with the manufacturer’s instructions. Genomic DNA was removed using the RNase-Free DNase Set (Qiagen, Hilden, Germany). Subsequently, the RNA integrity number (RIN) was assessed (mean ± SE = 8.66 ± 0.04) with the Agilent 2100 Bioanalyzer using the Agilent RNA 6000 Nano kit (Agilent Technologies, Waldbronn, Germany). Sequencing libraries were prepared using the TruSeq Stranded mRNA Sample Preparation kit (Illumina, San Diego, CA, USA) according to the manufacturer’s instructions. The quality of the DNA libraries was also determined by the Agilent Technologies 2100 Bioanalyzer and the Agilent DNA-1000 Chip kit (Agilent Technologies, Waldbronn, Germany). Concentration of the DNA libraries was quantified by the Qubit dsDNA HS assay kit (Invitrogen, Darmstadt, Germany). The cBot system (Illumina, San Diego, CA, USA) was used to generate clonal clusters, and sequencing was performed on the Illumina HiSeq 2500 sequencing platform with paired-end reads of 2 × 101 bp. The quality of pre- and post-processing data was assessed by the FastQC version 0.11.7 (http://www.bioinformatics.babraham.ac.uk/projects/fastqc/). The raw sequence files (fastq format) were preprocessed using TrimGalore version 0.5.0 to remove adapter-like sequences, and to trim low-quality reads (Q-score < 20) and short reads (< 30 bp). The resultant clean reads were then mapped to the reference genome Ssrofa11.1 (Ensembl release 98) using HISAT2 (version 2.1.0) [15]. Subsequently, the aligned reads were quantified using the HTSeq (version 0.11.2) [16]. The initial dataset contained 31,907 gene entries.

## Differential expression analysis

Prior to the analysis, pre-filtering was carried out to remove genes associated with fewer than eight samples with normalized counts greater than or equal to 5; this retained 14,809 available genes from the initial 31,907 gene entries. Principal component analysis (PCA) was performed based on variance-stabilizing transformed (VST) counts of all analyzed genes. Four outlier samples were identified and omitted from further analyses. Cell type enrichment analysis was performed with the xCell webtool [17], using transcripts per million (TPM) of all filtered genes. A t-distributed stochastic neighbor embedding (tSNE) plot was generated using the R package Rtsne version 0.15 [18] based on the enrichment scores of 64 cell types obtained from the xCell.

Differential gene expression analysis was conducted using the R package DESeq2 version 1.28.1 [19]. Three factors were included in the design of the statistical model: sex (male and female), GR genotype (AlaAla, AlaVal, and ValVal) [11], and treatment (CON, DEX, LPS, and LPS+DEX). Treatment effect was analyzed using the Wald test in five pairwise comparisons: DEX and vehicle groups (DEX VS CON), LPS and vehicle groups (LPS VS CON), LPS+DEX and vehicle groups (LPS+DEX VS CON), LPS+DEX and DEX groups (LPS+DEX VS DEX), as well as LPS+DEX and LPS groups (LPS+DEX VS LPS). Genes with a false discovery rate adjusted *p*-value (*q*-values) < 0.05 were considered to be significantly differentially expressed. A volcano plot was made using the R package EnhancedVolcano version 1.6.0 [20] to illustrate differentially expressed genes (DEGs). The heat map was plotted using the R package pheatmap version 1.0.12 [21] based on the log_2_ fold change (LFC) of the analyzed genes. A Venn diagram was created using the TBtools toolkit [22].

## Identification of functional modules and their hub genes

To study specific LPS and DEX functions in the context of their interplay, five modules (M1-M5) with different response patterns were identified based on significance and LFC. These modules comprised 4966 genes out of 8740 non-repetitive DEGs that were significantly regulated in at least one comparison. The criteria for defining each of the five functional modules are summarized in Supplementary Table 2. Following this, k-means clustering of gene expression profiles within each module was performed using the R package ComplexHeatmap version 2.4.3 [23]. Cytokines, chemokines, and their receptors were identified using the ImmPort cytokine registry [24]. GR targets involved in immune responses were identified by comparing with a gene list comprising genes shared by three libraries: GR-regulated genes from the database of Ingenuity Pathway Analysis (IPA; Qiagen, Hilden, Germany); GR targets revealed by binding site profiling studies from Harmonizome [25]; and immune genes from InnateDB [26]. For each module, protein–protein interaction networks were constructed within each module using the STRING database [27] and were then visualized using Cytoscape version 3.8.0 [28]. The top 30 hub genes displaying a high degree of connectivity were determined using the cytoHubba Cytoscape plugin [29]. Subsequently, the functional annotation of hub genes was performed with the ontology knowledgebase GO Biological Processes and Reactome Gene Sets using Metascape [30].

## Functional enrichment analysis

Enrichment analysis of canonical pathways, diseases, biological functions, and upstream regulators was conducted using the IPA to uncover directional regulation of signaling, biological consequences, and upstream regulatory events. For this purpose, *q*-value and LFC calculated by different comparisons were used for different modules: LPS VS CON and DEX VS CON were used for M1 and M2, respectively, to illustrate the influence of LPS and DEX; LPS+DEX VS LPS was used for M3 to highlight the anti-inflammatory effect of DEX; LPS+DEX VS DEX was used for M4 to determine potential events induced by LPS that may be involved in impaired DEX effect; LPS+DEX VS CON was used for M5 to reveal consequences of the additive or synergistic effects of LPS and DEX. Terms with *p*-values < 0.05 and with absolute z-scores ≥ 2 were considered to be significantly enriched and directionally regulated. Enrichment analysis with ontology sources GO Biological Processes was conducted using the Metascape to complement IPA results. The R package ggplot2 version 3.3.2 [31] and GraphPad Prism 8.2.1 (GraphPad Software, Inc., San Diego, CA) were used to visualize the results.

## Results

### Differential expression analysis

**Figure 1.**
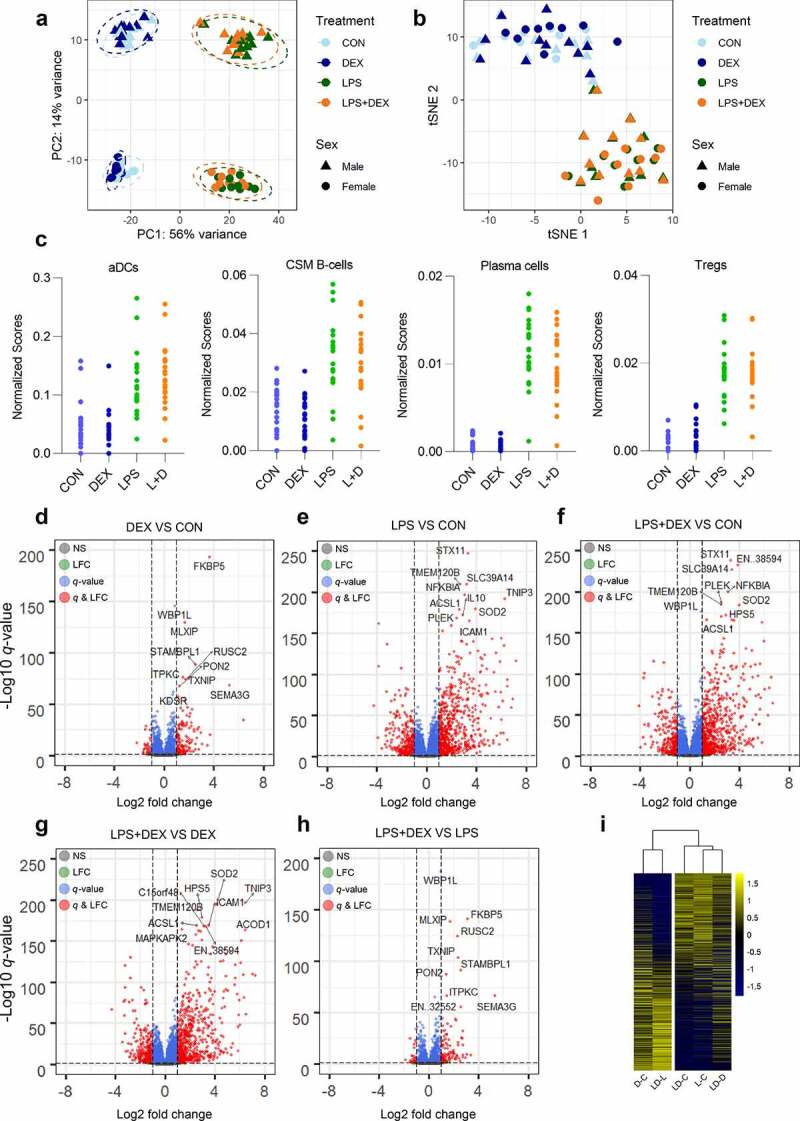
Differential expression analysis. (a) Principle component analysis of gene expression profiles using variance-stabilizing transformed (VST) counts of genes that passed filtering. (b) t-distributed stochastic neighbor embedding (t-SNE) plot of samples using enrichment scores of 64 cell types generated by cell type enrichment analysis via xCell webtool. TPM (transcripts per kilobase million) of genes that passed filtering were used for cell type enrichment analysis. (c) Enrichment scores of four immune cell types generated by cell type enrichment analysis. (d-h) Volcano plots of pairwise comparisons DEX VS CON (d), LPS VS CON (e), LPS+DEX VS CON (f), LPS+DEX VS DEX (g), and LPS+DEX VS LPS (h). Top 10 most significant genes in each comparison were determined by *q*-value and highlighted in the plot. (i) Heatmap constructed using LFC of genes in matched comparisons. Values were centered in the row direction. Abbreviation: CON, control; DEX: dexamethasone; LPS, lipopolysaccharide; aDCs, activated dendritic cells; CSM B-cells, class-switched memory B cells; Tregs, regulatory T cells; EN..38594, ENSSSCG00000038594; EN..32552, ENSSSCG00000032552

After filtering, a total of 14,809 genes were retained for the differential expression analysis (Supplementary Table 1). PCA revealed a primary separation of the samples by treatment type and a secondary separation of samples by sex (Figure 1a). Overall, the results showed that the treatment type shifted samples in the same direction regardless of sex (i.e. there is no obvious treatment by sex interaction). In contrast, cell type-specific responses were primarily due to the treatment, without an obvious effect of sex (Figure 1b). In addition, the cell-type enrichment scores indicate activation of regulatory T cell (Treg) signaling upon LPS stimulation (Figure 1c).

The five treatment comparisons (DEX VS CON, LPS VS CON, LPS+DEX VS CON, LPS+DEX VS DEX, and LPS+DEX VS LPS) yielded 2418 DEGs (1123 up- and 1295 downregulated), 6365 DEGs (3042 up- and 3323 downregulated), 6680 DEGs (3255 up- and 3425 downregulated), 5348 DEGs (2639 up- and 2709 downregulated), and 1812 DEGs (849 up- and 963 downregulated), respectively (Figure 1d-h; Supplementary Table 1).

*FKBP5*, a co-chaperone of GR, showed the most potent responsiveness to DEX, whereas *STX11*, implicated in the transport of LPS-activated TLR4 (Toll-like receptor 4) to the plasma membrane, showed the most potent responsiveness to LPS (Figure 1d, e). Several negative regulators of inflammation, such as *TNIP3* (*ABIN3), NFKBIA, IL10, SOD2*, and *ACOD1*, were strongly upregulated by LPS (Figure 1e, g). Clustering based on the LFC of DEGs revealed approximately inverse directions of LPS and DEX effects (Figure 1i).

## Distinct biological meanings of typical genes in functional modules

**Figure 2.**
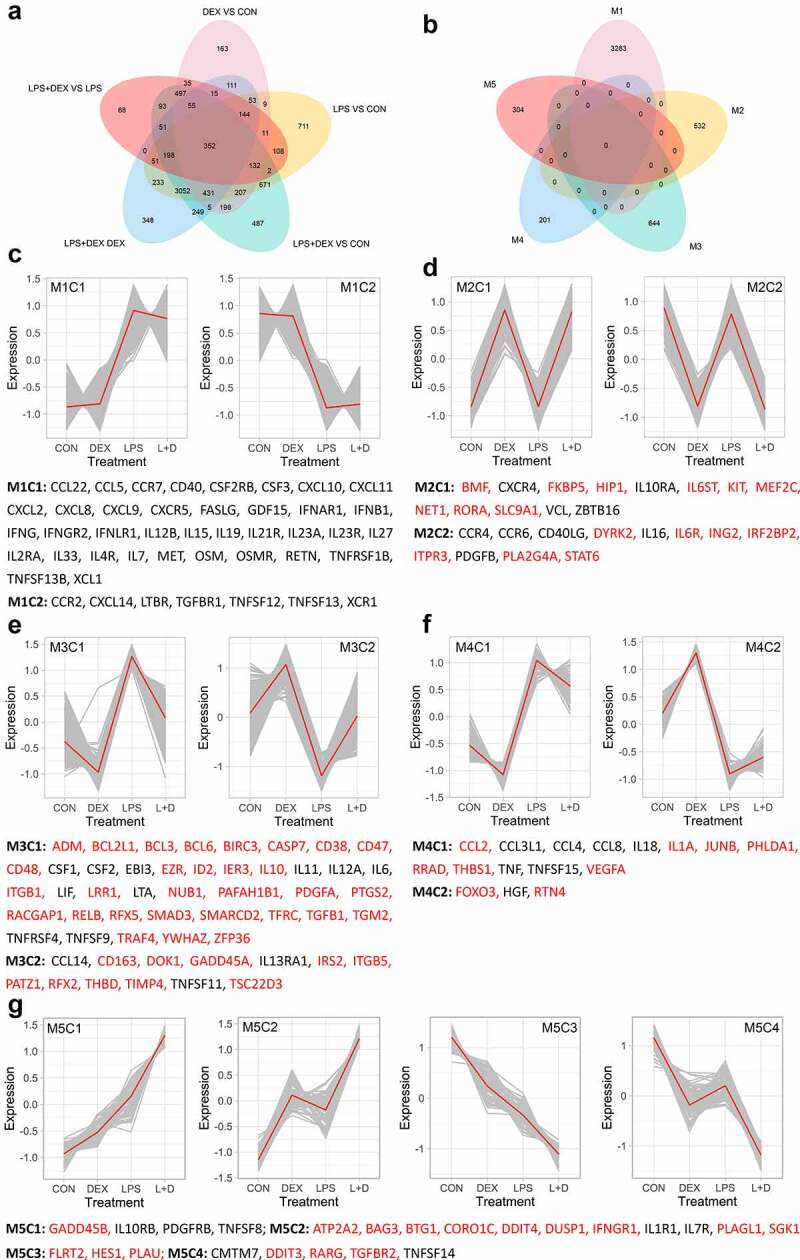
Functional modules and gene expression patterns. (a) Venn diagram illustrating unique and shared DEGs among five pairwise comparisons. (b) Venn diagram illustrating unique and shared DEGs among five functional modules. (c-g) K-means clustering of gene expression profiles of five functional modules M1 (c), M2 (d), M3 (e), M4 (f), and M5 (g). Cytokines, chemokines and their receptors (for M1-M5, identified using the ImmPort [24]) as well as GR targets implicated in immune responses (for M2-M5, in red, identified using the InnateDB [26], Harmonizome [25], and Ingenuity Pathway Analysis (IPA)) were indicated below corresponding modules

To study specific LPS and DEX functions in the context of their interplay, five functional modules (M1-M5; Supplementary Table 2) with different response patterns were identified from DEGs that were significantly regulated by at least one stimulus. A total of 4966 genes, comprising almost all DEGs that were shared by all comparisons (350 out of 352, with the exception of *NIBAN2* and *TRIB3* without notable response patterns), could be assigned into a module (Figure 2a, b; Supplementary Table 1).

The genes allocated to M1 (n = 3285) were significantly regulated by LPS but not by DEX. Inversely, genes in M2 (n = 532) were regulated by DEX but not by LPS. Genes in module M3 (n = 644) were oppositely regulated in LPS VS CON and LPS+DEX VS LPS, while the genes in M4 (n = 201) were regulated in the opposite direction by LPS (LPS VS CON and LPS+DEX VS DEX) and DEX VS CON. In addition, these genes showed no significant response in LPS+DEX VS LPS, which implies that DEX effect on their expression was blunted by the LPS co-treatment under the applied experimental conditions. In M5 (n = 304), it was found that the genes were affected by LPS and DEX in either an additive or synergistic way. In Figure 2 (c-g) functionally important members of the modules, including cytokines, chemokines, and their receptors (M1-M5), as well as the GR targets involved in immune responses (M2-M5) are displayed.

Besides a subset of pro-inflammatory cytokines and chemokines upregulated by LPS, module M1 also encompasses several positive regulators of immune response downregulated by LPS, including *CCR2, CXCL14, LTBR, TNFSF12, TNFSF13, XCR1, C5AR1, C5AR2, MAP3K3, MAP4K3, MAP4K5, FOS, IRF5, KLF6, TLR4, TLR5*, and *TLR8* (Figure 2c; Supplementary Table 1). This finding suggests that parallel to the pro-inflammatory response LPS triggered a compensatory, homeostatic anti-inflammatory program. This proposition is corroborated by the upregulation of several negative regulators of immune response assigned to module M1, including *ANXA1, ANPEP, ACOD1, DUSP16, ETV3, IRF4, SOD2*, and *STAT3*, by LPS (Supplementary Table 1).

Module M2 comprised several immune genes, such as *IL16* and *CD40LG*, that were downregulated by DEX, but did not respond to LPS under our experimental condition (Figure 2d). The genes in module M3 characterize the anti-inflammatory function of DEX, which was indicated, for example, by the inhibition of pro-inflammatory *RELB* and *IL6* and upregulation of anti-inflammatory *ADORA3, CD163, DOK1*, and *TSC22D3* in LPS+DEX VS LPS (Figure 2e; Supplementary Table 1). Unlike module M3, several cytokines, chemokines, and their mediators assigned to module M4 were not efficiently regulated by DEX when co-treated with LPS; this included *TNF, IL1A, IL18, CCL2, CCL4, CCL8*, and *IRF3*. Thus, the genes in module M4 will allow a better understanding of the causes and consequences of reduced DEX sensitivity in the context of the pro-inflammatory response triggered by LPS (Figure 2f; Supplementary Table 1).

For the genes in M5 that showed an additive or synergistic effect of LPS and DEX, two main biological meanings can be deduced; firstly, these represent anti-inflammatory function of LPS as shown by the induction of the anti-inflammatory *TNFAIP3* and *DUSP1*, and inhibition of the pro-inflammatory *TNFSF14* (Figure 2g; Supplementary Table 1); secondly, DEX also exhibits pro-inflammatory effects as evidenced by the activation of pro-inflammatory *IL1R1, IL1RAP, IRAK2, CD14, MYD88, CD80, TNFSF8, IL7R, JAK1*, and *STAT5B* and the inhibition of anti-inflammatory *NKIRAS1* and *NRROS* (Figure 2g; Supplementary Table 1). These results will help to determine the priming effects of stress-induced GCs, which could subsequently enhance the vulnerability for subsequent inflammatory stimuli.

## Typical genes of functional modules have high connectivity

**Figure 3.**
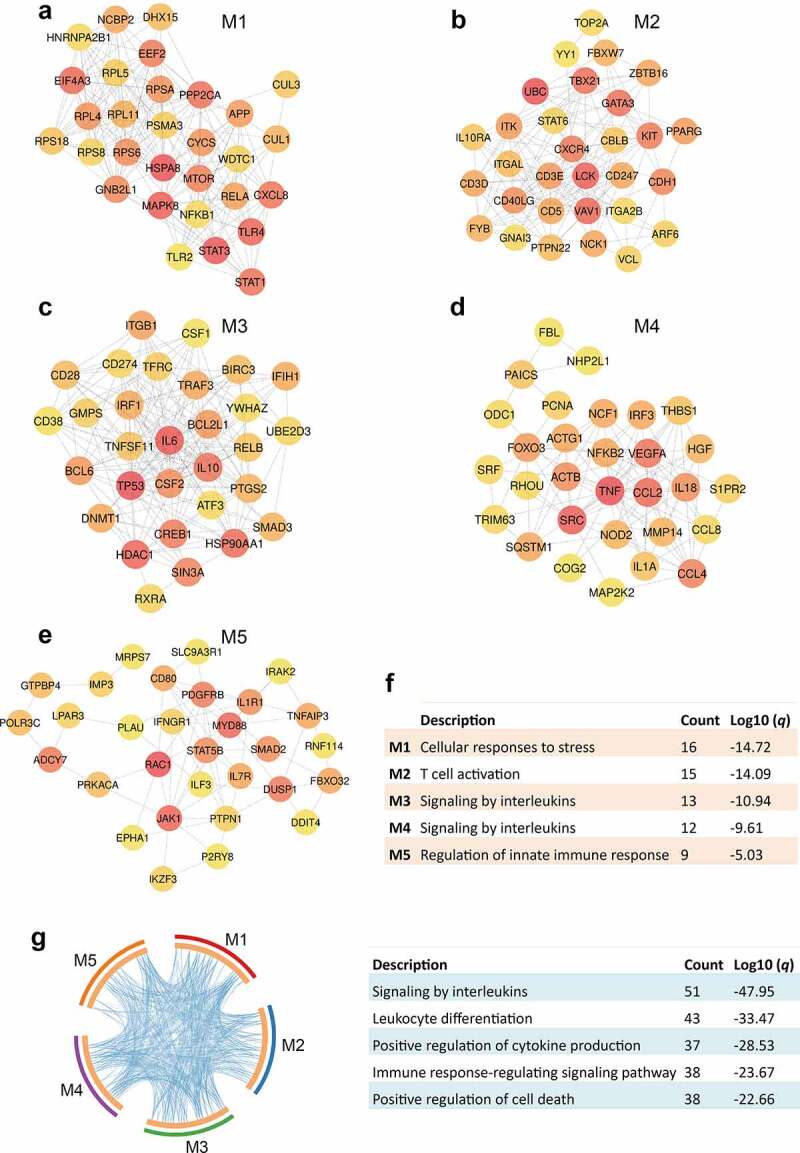
Identification and annotation of hub genes of functional modules. (a-e) Protein–protein interaction (PPI) networks of top 30 hub genes with high connectivity within modules M1 (a), M2 (b), M3 (c), M4 (d), and M5 (e). PPI networks were constructed by the STRING resource within modules [27] and top 30 central elements of each network were identified and visualized by the Cytoscape plugin cytoHubba [28,29]. Connectivity was correlated with color of circles where red indicates higher degree and yellow indicates lower degree. (f) The most significant biological function of hub genes of each module. (g) Overlap of functions of hub genes of different modules. The blue lines in the Circos plot linked genes annotated by the same functional term. Top five functional terms that were significantly enriched for all modules are shown on the right panel. For (f) and (g), annotation was performed with ontology sources GO Biological Processes and Reactome Gene Sets using the Metascape [30]

Protein–protein interaction networks were constructed within modules, which then allowed the identification of the top 30 hub genes that showed a high degree of connectivity (Figure 3). In line with LPS-induced inflammation, genes involved in NF-κB and MAPK cascades, such as *RELA, NFKB1, STAT1*, and *MAPK8*, were identified as hub genes in M1. Notably, the pleiotropic TF *STAT3* involved in the IL-10 mediated anti-inflammatory response was also included as a hub gene in M1 (Figure 3a). Functional annotation revealed that hub genes in this module were implicated in cellular responses to stress (figure 3f). The most marked hub genes in M2 were associated with T cell functions, including *CD40LG, LCK, TBX21, GATA3, CD5, CD3D, CD3E*, and *CD247* (*CD3Z*) (Figure 3b; Figure 3f). In M3, DEX caused the downregulation of inflammation-related hub genes such as *RELB* and *IL6* (Figure 3c). Furthermore, *TNF, IL1A, IRF3, NFKB2*, and *MAP2K2* in M4 showed high connectivity, suggesting their fundamental role in counteracting the effects of DEX on pro-inflammatory responses (Figure 3d). Signaling by interleukins was enriched for both M3 and M4 (Figure 3f). The hub genes in M5 evidence the previously discussed biological meanings of the additive or synergistic effects. This is shown by the involvement of *DUSP1* and *TNFAIP3* in the LPS-induced anti-inflammatory response and by *IL1R1, IRAK2, MYD88, CD80, IL7R, JAK1*, and *STAT5B* in the DEX-induced pro-inflammatory response (Figure 3e). These genes were enriched for functions related to regulation of innate immune response (Figure 3f). Certain functional themes were found to be enriched for the hub genes of all modules, such as signaling by interleukins and leukocyte differentiation (Figure 3g).

## Canonical pathways, biological consequences, and upstream regulators of functional modules

**Figure 4.**
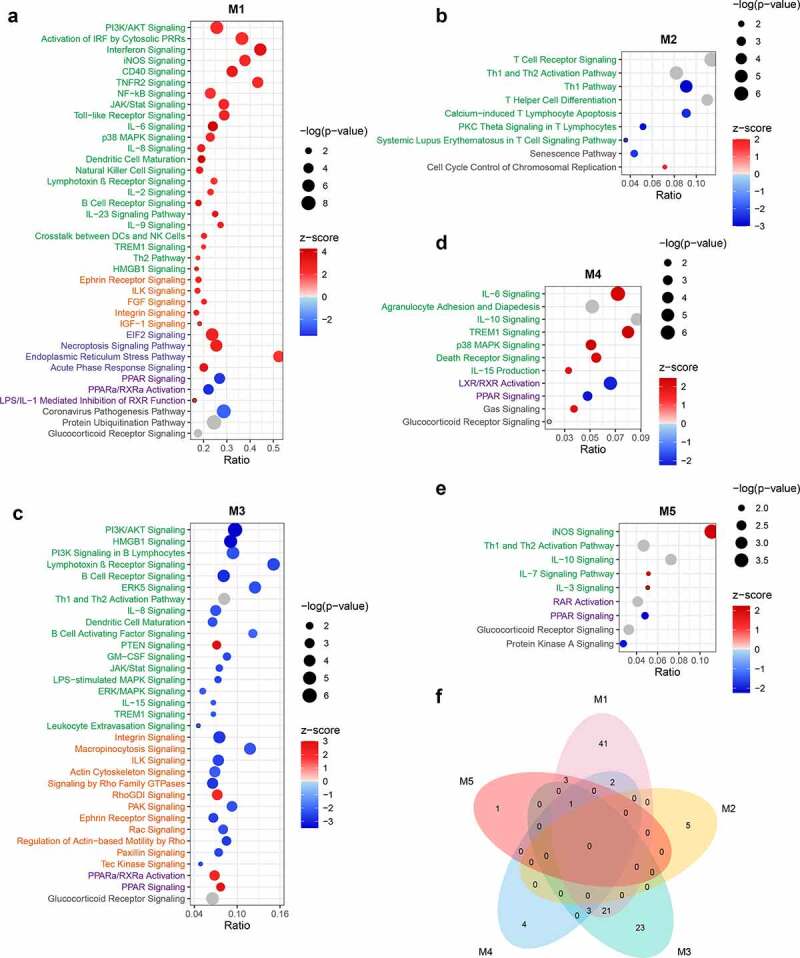
Canonical pathways enriched for functional modules. (a-e) Bubble diagram illustrating representative canonical pathways for modules M1 (a), M2 (b), M3 (c), M4 (d), and M5 (e). Enrichment was carried out with the IPA. The significance of terms was correlated with bubble size where large size indicates smaller *p*-values and all shown terms had *p*-values < 0.05. Enrichment z-scores were indicated by color of bubbles where red indicates z-score > 0 and blue indicates z-score < 0. Terms with unavailable z-scores were indicated in gray. The name of terms belonging to different categories was indicated in different color where green indicates immune response, orange indicates cytoskeleton and cell motility, blue indicates stress response and necroptosis, and purple indicates PPAR-related signaling. (f) Venn diagram illustrating unique and shared canonical pathways among five modules. Only terms with absolute z-scores ≥ 2 were used for the diagram

**Figure 5.**
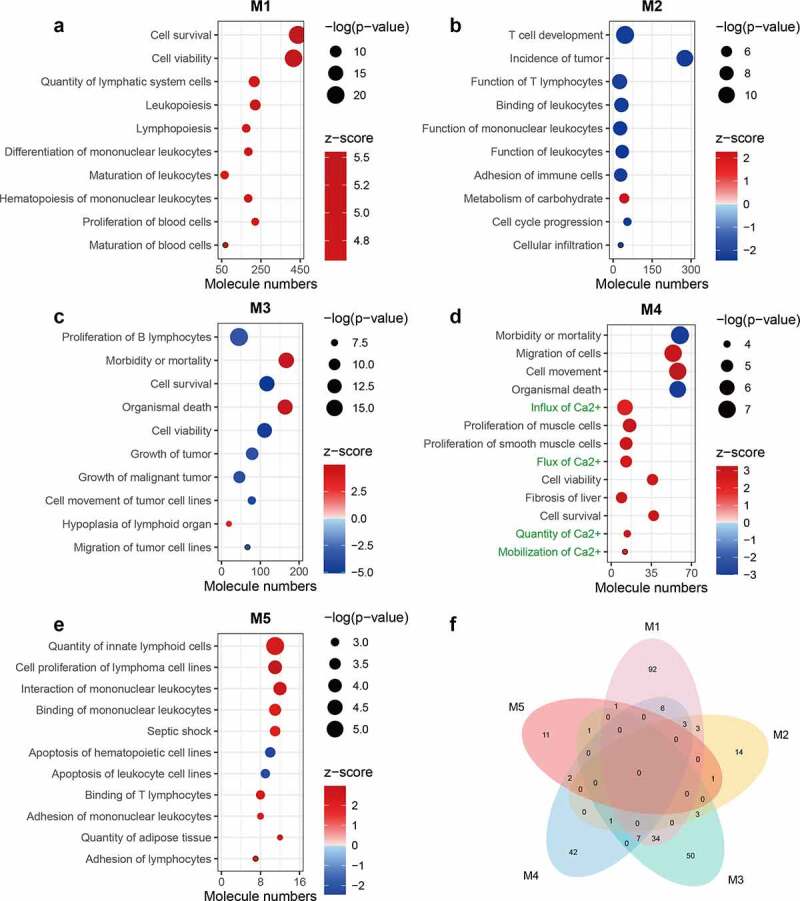
Biological consequences of functional modules. (a-e) Bubble diagram illustrating representative diseases or biological functions for modules M1 (a), M2 (b), M3 (c), M4 (d), and M5 (e). Enrichment was carried out with the IPA. The significance of terms was correlated with bubble size where large size indicates smaller *p*-values and all shown terms had *p*-values < 0.05. Enrichment z-scores were indicated by color of bubbles where red indicates z-score > 0 and blue indicates z-score < 0. Calcium-related terms in (d) were indicated in green. (f) Venn diagram illustrating unique and shared terms among five modules. Only terms with absolute z-scores ≥ 2 were used for the diagram

**Figure 6.**
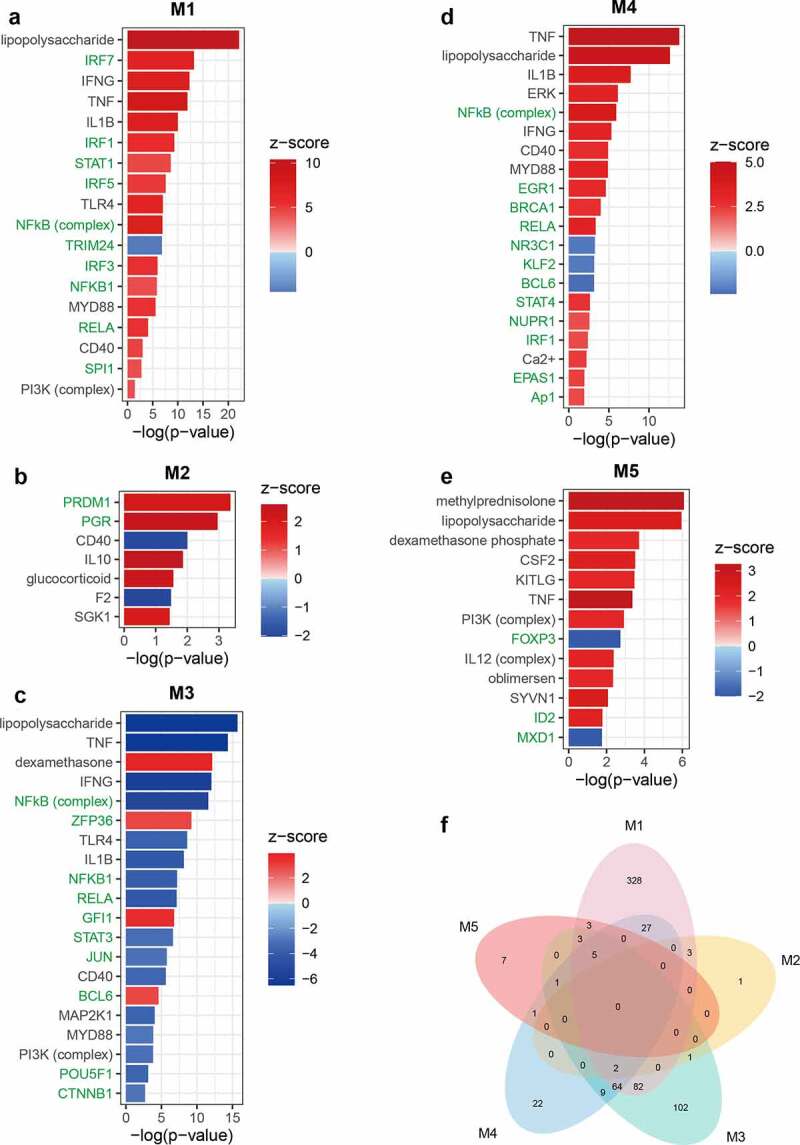
Potential upstream regulators enriched for functional modules. (a-e) Bar diagram illustrating representative upstream regulators for modules M1 (a), M2 (b), M3 (c), M4 (d), and M5 (e). Enrichment was carried out with the IPA. Enrichment z-scores were indicated by color of bars where red indicates z-score > 0 and blue indicates z-score < 0. The name of transcription factors (TFs) was indicated in green. For M2, M4, and M5, all predicted upstream TFs were shown in the figure and for M1 and M3, only top 10 upstream TFs with high absolute z-scores were shown due to the large number of predicted terms. (f) Venn diagram illustrating unique and shared upstream regulators among five modules. Only terms with absolute z-scores ≥ 2 were used for the diagram

The module M1 presents the activation of a series of pathways involved in the initiation, signal transduction, and effector stages of inflammation. This was consistent with the LPS-induced activation of the inflammatory response shown in the LPS VS CON comparison (Figure 4a; Supplementary Table 3). Activation of the IRF and NF-κB signaling pathways suggested that both MYD88-dependent and -independent signaling were triggered by LPS. Activation of necroptosis signaling was also identified, which is in line with the upregulation of its key mediator *MLKL*. The substantial activation of immune responses by the identified genes was supported by the enrichment of GO terms linked with the regulation of the innate immune response (Supplementary Table 4). In addition, a set of pathways involved in cytoskeletal reorganization were activated in M1 (Figure 4a). This is consistent with the predicted activation of IPA terms related to proliferation, maturation, survival, and viability of leukocytes (Figure 5a; Supplementary Table 5). Despite the dramatic activation, rare pathways were inhibited in M1 such as anti-inflammatory PPAR signaling (Figure 4a). These results indicate a predominantly pro-inflammatory state in porcine PBMCs triggered by LPS application. In M1, the predicted activation of upstream pro-inflammatory TFs by IPA, such as *IRF7, STAT1, NFKB1*, and *RELA*, corresponds to the observed upregulation of their expression (Figure 6a). Furthermore, several negative regulators of the immune response, such as *STAT3* and *NFKBIA* (z-score = 1.855) were predicted or tended to be activated for M1. This corresponded with their increased expression following LPS treatment, although *NFKBIA* itself was not assigned a module (Supplementary Table 1; Supplementary Table 6).

The most notable insight from M2 (DEX VS CON) was the inhibition of T cell signaling in response to DEX (Figure 4b; Figure 5b). *CD40*, a key mediator conveying signals between T cells and other immune cells was predicted to be inhibited in M2 (Figure 6b). This is matched by the reduced expression of its ligand *CD40LG* (Figure 2d). As the M2 genes failed to respond to LPS in this particular study, these results suggest a constitutive inhibition of T cell function by DEX.

Many pathways and predicted upstream regulatory events enriched for M3 showed opposite directions compared with M1. This was seen for the inhibition of B cell receptor signaling (Figure 4c) and for the upstream regulator NF-κB (Figure 6c). The inhibition of B cell signaling was supported by the predicted inhibition of the biological consequence proliferation of B lymphocytes (Figure 5c). In module M3, DEX inhibited a set of pathways associated with cytoskeletal remodeling, suggesting that DEX might directly influence cytoskeleton-mediated immune cell function (e.g. phagocytosis and trafficking [32]) (Figure 4c). Furthermore, M3 highlighted the role of Rho family of GTPases, a type of well-known molecular switches, in controlling inflammation caused by DEX (Figure 4c). These results are supported by the enrichment of GO terms related to small GTPase mediated signal transduction and actin cytoskeleton organization (Supplementary Table 4).

The enrichment of p38 MAPK signaling in M4 (LPS+DEX VS DEX) was found by both IPA and GO analyses (Figure 4d; Supplementary Table 4). Several biological consequences related to the influx of Ca^2+^ were predicted to be activated exclusively in M4 (Figure 5d, f; Supplementary Figure 1a). These predictions were supported by the upregulation of *ORAI1* and the predicted activation of Ca^2+^ as an upstream regulator (Supplementary Table 1; Figure 6d). *TNF* has been identified as the most prominent upstream regulator for M4, which corresponds with its upregulation and assignment to this module. *KLF2* was predicted to be inhibited, however it did not show a clear direction for any other modules (Figure 6d, f). *KLF2* is a GC-inducible anti-inflammatory TF that can reduce the LPS-stimulated cytokine production by inhibiting the transcriptional activity of NF-κB and AP-1 [33]. However, in our study, *KLF2* itself did not respond to DEX (Supplementary Table 1). This observation, together with predicted inhibition of *KLF2* in M4, implies that the inability of DEX to activate *KLF2-*mediated transcriptional response contributes to the weakened DEX-responsiveness of a subset of genes.

In M5, strong activation of the iNOS signaling was predicted (Figure 4e). Cytokine-induced iNOS promotes the pathogenesis of septic shock due to excessive production of NO [34]. In combination with the predicted activation of septic shock (enriched only for M5), these results suggest that DEX-mediated pro-inflammatory action might result in severe pathological consequences, such as sepsis (Figure 5e, f; Supplementary Figure 1b). In line with the downregulation of *PRKACA* that encodes the catalytic subunit α of protein kinase A (PKA), PKA signaling was predicted to be inhibited for M5 (Figure 4e). It could block NF-κB transcription via interaction with p65 and could potentially improve GR function in both ligand-dependent and -independent manners [5]. Thus, it was suggested that the pro-inflammatory action of DEX is linked to the impaired inhibition of NF-κB signaling and the alteration of GR function. This hypothesis was supported by the inhibition of *NKIRAS1* and by the enrichment of GR signaling (Supplementary Table 1; Figure 4e). The pro-inflammatory effect of DEX could also be implicated in the dysfunction of Tregs since *FOXP3*, a critical TF controlling the development and function of Tregs [35] was predicted to be inhibited (Figure 6e). However, in our study *FOXP3* itself was not regulated by the treatment at the transcriptional level. In addition, we observed that many pathways were shared by more than one module, in particular, PPAR signaling, which was enriched for all modules except M2 (Figure 4f).

## Discussion

In this study, we identified extensive transcriptional responses as a result of both LPS and DEX applications. Based on these response patterns, five functional modules were established. Two major findings emerged from bioinformatic analysis of the modules; firstly, although LPS triggered predominantly pro-inflammatory responses, it concurrently induced an anti-inflammatory response. This study clearly depicted this anti-inflammatory feedback through a subset of LPS-repressed pro-inflammatory genes involved in cytokine and chemokine activities (*CCR2, CXCL14, LTBR, TNFSF12, TNFSF13*, and *XCR1*), complement system (*C5AR1* and *C5AR2*), TLR signaling (*TLR4, TLR5*, and *TLR8*), MAPK cascades (*MAP3K3, MAP4K3*, and *MAP4K5*), and regulation at the transcriptional level (*FOS, IRF5*, and *KLF6*). The downregulation of *TLR*s by LPS is described in a previous expression array study, where stimulation of porcine PBMCs by LPS for 24 hours represses expression of *TLR6* and *TLR8* [36]. In our study, the expression of *TLR6* was not changed by LPS. Unlike the above *TLRs, TLR2* was upregulated by LPS and identified as a hub gene in M1. *TLR2* is a target of TF *RUNX1* [37], and the latter was also upregulated by LPS in our study. *RUNX1* is highly expressed in porcine PBMCs and is crucial for T and B cell development and activation [37]. Three *RUNX1* targets, including *TLR2, LCK*, and *VAV1*, were upregulated by LPS in porcine PBMCs after treatment for 6 hours [37]. Unlike *TLR2*, in our study *LCK* and *VAV1* were allocated to M2C2; they were repressed by DEX but did not respond to LPS under the applied experimental conditions.

Furthermore, this response comprised a series of LPS-induced anti-inflammatory molecules; in M1 this included *ANXA1, ANPEP, ACOD1, DUSP16, ETV3, IRF4, SOD2*, and *STAT3*, and outside of M1 it included *TNIP3, TNFAIP3, NFKBIA, IL10*, and *DUSP1*. Many of these genes suggest a negative regulation of the NF-κB cascade. *TNIP3* binds to TNFAIP3, a dual-function ubiquitin-editing enzyme, and inhibits NF-κB activation induced by TNF and IL-1 [38]. TNFAIP3 suppresses NF-κB activity through the removal of Lys-63-linked ubiquitin chains from, and/or adding degradation-inducing Lys-48 ubiquitin chains, to protein kinase RIPK1 upstream of IKK activation [39]. Upregulation of *ANXA1* and *NFKBIA*, negative regulators of NF-κB [2], provides additional evidence for the inhibition of NF-κB signaling. LPS-induced upregulation of *SOD2* has been detected previously in porcine PBMCs [36]. *SOD2* belongs to the superoxide dismutase family that can inhibit lipid peroxidation-based release of inflammatory mediators such as prostaglandins, thromboxanes, and leukotrienes [40]. *SOD2* also hampers NF-κB activity and reduces TNF-α and IL-1β levels in LPS-activated microglia [41]. In addition to reducing the expression of positive mediators in MAPK signaling, LPS further upregulated the dual-specificity phosphatase 1 (*DUSP1*), which could inhibit inflammation through dephosphorylation and subsequent inactivation of MAPKs [2].

LPS-initiated immunosuppression might also be involved in the activation of IL-10/STAT3 signaling. IL-10 is a prominent anti-inflammatory cytokine with the ability to repress several LPS-inducible genes and antigen-presenting markers [42]. IL-10 stimulates activation of STAT3, which is necessary for the IL-10-mediated anti-inflammatory functions [43]. IL-10 can also promote the TLR-induced expression of *ZFP36* to reduce the mRNA stability of cytokines such as IL-1β and TNF-α; this is achieved by targeting AU-rich elements in the 3ʹ untranslated region [44]. The destabilization of mRNA by *ZFP36* could be enhanced by *DUSP1* via dephosphorylation of p38 MAPK. In turn, *DUSP1* could be induced by IL-10 [44]. Thus, the observed enhanced expression of *IL10, STAT3, DUSP1*, and *ZFP36* by LPS clearly supported the onset of this anti-inflammatory program. Furthermore, several negative mediators of the immune response downstream of the IL-10/STAT3 signaling were upregulated by LPS, such as *DUSP16* and *ETV3* [44].

An additional key theme in this study was the crosstalk between components of immune responses and GR signaling. This was shown through DEX-mediated constitutive inhibition of T cell signaling, DEX-mediated inhibition of inflammation, LPS interfering with the anti-inflammatory action of DEX, and DEX-mediated pro-inflammatory action.

Although GCs influence almost all immune cell types [45], genes that were shown to be significantly regulated by DEX but showed a lack of responsiveness to LPS were correlated with the inhibition of T cell functions; this was evidenced by canonical pathways and biological consequences enriched in M2. Accordingly, several hub genes involved in T cell functions were downregulated by DEX in M2, including *CD247, CD3D, CD3E, CD40LG, LCK*, and *TBX21*. Specifically, *CD247, CD3D*, and *CD3E* constitute part of the T cell receptor (TCR)-CD3 complex, which plays a vital role in the recognition of signals from antigen-presenting cells (APCs) [46]. The CD3 chains all possess immunoreceptor tyrosine-based activation motifs (ITAMs) that can be phosphorylated by *LCK*, a member of the Src family of protein tyrosine kinases. Consequently, this activates immune signaling [46]. Thus, inhibition of CD3 molecules and *LCK* suggests DEX-mediated constitutive inhibition of TCR/CD3 signaling. The inhibition of T cell and APC engagement by DEX was corroborated by the downregulation of *CD40LG*. The T cell expression of *CD40LG* mediates immune responses by interacting with *CD40* expressed on APCs and B cells [47]. Therefore, inhibition of *CD40LG* may block signal transduction between T cells and other immune cells. *TBX21* is a lineage-specific TF expressed by Th1 cells; it was found to be downregulated by DEX in the current study. The inhibition of *TBX21* function by GCs occurs due to a reduction in mRNA and protein levels, but it is also a result of diminished binding of *TBX21* to DNA [45].

DEX-mediated inhibition of inflammation was evident in this study through the inhibition of pro-inflammatory genes, alongside increased expression of anti-inflammatory genes including *TSC22D3, ADORA3, CD163*, and *DOK1* [2]. *TSC22D3*, also known as *GILZ*, inhibits PI3K/AKT and MEK/ERK signaling through Ras and Raf-1 interactions [2]. Therefore, the upregulation of *TSC22D3* is consistent with significant inhibition of PI3K/AKT and ERK signaling as predicted by IPA. *TSC22D3* also inhibits NF-κB and AP-1 through the interplay with p65, and c-Fos and c-Jun subunits, respectively [2]. This is supported by the predicted inhibition of upstream regulators NF-κB and JUN in this study. *ADORA3* is another GC-dependent anti-inflammatory gene with possible PI3K and NF-κB interactions [48]. It is highly expressed in immune cells, including PBMCs; this distinguishes it as an important therapeutic target for many immune diseases [48]. Moreover, *DOK1*, an inhibitory adaptor protein with the ability to suppress MAPK cascades, was upregulated by DEX [49]. *CD163* exerts anti-inflammatory action by eliminating hemoglobin-haptoglobin complexes [50]. These findings demonstrate the fundamental role of DEX-inducible genes in terms of the anti-inflammatory action of DEX in porcine PBMCs.

We observed a distinct attenuation of the DEX-mediated anti-inflammatory effect due to LPS, which parallels the upregulation of *TNF*. Pretreatment with TNF-α was shown to reduce DEX-mediated inhibition of IL-6 in human whole-blood cell cultures [51]. TNF-α can impair GR-mediated transcriptional regulation by reducing the accessibility of the transcriptional cofactor p300 to GR; this was conveyed by NF-κB via sequestration of p300 [4]. The acetyltransferase activity of p300 is indispensable for GR-mediated transcription [52]. In addition, p300 can also serve as a scaffold for the recruitment of other cofactors involved in GR functions [53]. Access of GR to p300 facilitates the interaction between GR and the transcription initiation complex and ensures the transduction of GC signal to RNA polymerase II [54]. The cytokine *IL1A* may also counteract the DEX anti-inflammatory action, which could inhibit DEX-induced GR translocation from cytoplasm to nucleus. Consequently, there would be decreased transcriptional activity of GR, paralleled by p38 MAPK activation [5]. Different from “master” cytokines *TNF* and *IL1A* initiating the earliest inflammatory response, the upregulation of “secondary” cytokines such as *IL6* and *IL10* by LPS was effectively inhibited by DEX in the present study [55].

LPS recognition by TLR4 activates two distinct downstream pathways, MYD88-dependent signaling which results in the activation of NF-κB and MAPK, and MYD88-independent signaling, which results in the activation of interferon regulatory factors *IRF3* and *IRF7* [56]. *IRF3*, showing high connectivity in M4, could potentially compete with GR for binding to its dual-function coregulator *GRIP1* [57]. This could result in decreased accessibility of *GRIP1* to GR. Certain p160 family members, such as *SRC1* and *RAC3*, act only as coactivators, while *GRIP1* also potentiates GR-mediated repression [58]. A previous study found that deficiency in *GRIP1* enhanced LPS-induced inflammation and reduced GR-mediated repression of NF-κB signaling in mice [59]. Moreover, activation domains (AD1 and AD2) of *GRIP1* have been shown to contribute to the recruitment of p300 [58]. These results suggest that the activation of MYD88-independent TLR4 signaling could possibly impair GRIP1-dependent GR-mediated anti-inflammatory action.

The results of this study indicated the potential involvement of Ca^2+^ signaling in the attenuation of the anti-inflammatory effect of DEX by LPS. This is supported, for instance, by the observed upregulation of *ORAI1* by LPS. ORAI1 is a subunit of the Ca^2+^ release-activated Ca^2+^ channel (CRAC) that plays a key role in the store-operated Ca^2+^ entry (SOCE) in T cells [60]. The induction of genes involved in Ca^2+^ pathways by LPS has been observed in porcine PBMCs [36]. However, the role of Ca^2+^ in the LPS-induced GC resistance has yet to be established. We hypothesize a mechanism involving reduced p300 availability caused by the activation of cyclic adenosine monophosphate (cAMP) signaling by Ca^2+^ and *ORAI1* [60]. Previous research has shown that activation of cAMP signaling can promote the degradation of p300 in human lung cancer cells [61]. On the other hand, Ca^2+^ positively regulates LPS-induced inflammation in macrophages in a dose-dependent manner, alongside ERK1/2 signaling activation [62]. Thus, Ca^2+^ signaling could also potentially promote LPS-induced diminishment of DEX effects, by regulating cytokine production. The direction of the regulation of genes in M4 meant that Ca^2+^ signaling was inhibited for DEX VS CON but not for LPS+DEX VS LPS. Essentially, DEX can reduce intracellular Ca^2+^ levels through a non-genomic action [63]. Thus, the enrichment of Ca^2+^ signaling in M4 implies possible impairment of DEX-mediated non-genomic effects caused by LPS.

GCs are typically described as anti-inflammatory agents, but emerging evidence suggested that they also exert pro-inflammatory effects. However, the mechanisms involved are not yet well understood [64]. In this study, we identified a series of molecules regulated by DEX that are likely to be responsible for its pro-inflammatory actions. These molecules indicated activation of TLR, NF-κB, iNOS, and IL-1 signaling. These included for instance *CD14* and *MYD88*, which play a vital role in the recognition of LPS and activation of TLR4 signaling. *CD14* binds to the LPS-binding protein (LBP)/LPS aggregate and facilitates the LPS transfer to MD2/TLR4 complex. As a result, MYD88-dependent signaling and NF-κB cascade are activated [65]. The involvement of NF-κB was supported by the reduced expression of its inhibitors *NKIRAS1* and *NRROS. NKIRAS1* negatively regulates NF-κB activity by preventing degradation of IκBβ, the inhibitory IκB protein [66]. In turn, *NRROS* inhibits NF-κB activation mediated by TLR4 [67]. Furthermore, the nitric oxide synthase iNOS is inducible by inflammatory stimuli, which promotes NO production and NOX-mediated ROS generation to benefit the host defense [68]. Excessive NO production downstream of cytokine-induced iNOS promotes the pathogenesis of septic shock [34], which corresponds with the observed upregulation of *CD80* [69] by DEX in this study, and the predicted activation of septic shock for M5. In addition, the pro-inflammatory effect of DEX was supported by the upregulation of *IL1R1, IL1RAP*, and *IRAK2*, three key subunits of a functional complex essential for IL-1 signaling [70].

In this study, blood samples used to isolate PBMCs were collected from pigs at exsanguination after electrical stunning. It has been reported that *in vitro* bovine IFN-γ response to tuberculosis antigen can be inhibited when collecting blood at exsanguination after electrical stunning [71]. Nevertheless, here in porcine PBMCs, both *IFNG* expression and interferon signaling were highly activated by LPS. It should also be noted that frozen rather than fresh PBMCs were used in this study. Despite the wide application of frozen PBMCs in immune research [72,73], the potential influence of cryopreservation should be considered when interpreting results of the current study.

Overall, our study provides a comprehensive overview of the responses triggered by LPS and DEX exposure in porcine PBMCs. Here, we show a novel analysis of the crosstalk between GR signaling and inflammatory pathways on a genome-wide scale in pigs. Regarding the mechanisms of pro-inflammatory pathways counteracting GR signaling, and the priming effects of DEX on pro-inflammatory genes, our findings have important implications for advancing animal health and progressing the application of GC-based drugs. We also derived novel hypotheses based on this study, such as the role of calcium signaling in the impairment of DEX functions, which deserve further investigation in the future.

## Supplementary Material

Supplemental Material

## Data Availability

The RNA-Seq dataset was submitted to the ArrayExpress repository (https://www.ebi.ac.uk/arrayexpress, accession number E-MTAB-9808) at EMBL-EBI.
