# Supplementary material for: Transcriptome analysis of porcine PBMCs reveals lipopolysaccharide-induced immunomodulatory responses and crosstalk of immune and glucocorticoid receptor signaling
Source: Virulence. 2021 Jul 21;12(1):1808–24. doi: 10.1080/21505594.2021.1948276 (PMC8296968; doi:10.1080/21505594.2021.1948276)
Supplement: Supplemental Material [file KVIR_A_1948276_SM5938.zip › supplementary/Supplementary_Table_2.docx]

**Supplementary Table 2. Criteria for defining the five functional modules.**

| **Modules** | **Criteria** |
| --- | --- |
| M1 | 1. LPS VS CON *q* < 0.05  2. LPS+DEX VS DEX *q* < 0.05  3. DEX VS CON *q* > 0.05  4. LPS+DEX VS LPS *q* > 0.05 |
| M2 | 1. DEX VS CON *q* < 0.05  2. LPS+DEX VS LPS *q* < 0.05  3. LPS VS CON *q* > 0.05  4. LPS+DEX VS DEX *q* > 0.05 |
| M3 | 1. LPS VS CON *q* < 0.05  2. LPS+DEX VS LPS *q* < 0.05  3. Log2 fold changes (LFCs) of LPS VS CON and LPS+DEX VS LPS show opposite directions |
| M4 | 1. DEX VS CON *q* < 0.05  2. LPS VS CON *q* < 0.05  3. LPS+DEX VS DEX *q* < 0.05  4. LPS+DEX VS LPS *q* > 0.05  5. LFCs of DEX VS CON show opposite directions with LPS VS CON and LPS+DEX VS DEX |
| M5 | 1. LPS+DEX VS LPS *q* < 0.05  2. LPS+DEX VS DEX *q* < 0.05  3. LPS+DEX VS CON *q* <0.05  4. LFCs of DEX VS CON, LPS VS CON, LPS+DEX VS CON, LPS+DEX VS LPS, and LPS+DEX VS DEX show the same direction |
